# Anomalous Differences Between the Global Lung Function Initiative 2023 and 2012 Spirometry Reference Values

**DOI:** 10.1016/j.chest.2025.06.005

**Published:** 2025-06-17

**Authors:** Brian L. Graham, Veronica Marcoux, Yet H. Khor, Allan L. Coates

**Affiliations:** a Division of Respirology, Critical Care and Sleep Medicine, University of Saskatchewan, Saskatoon, SK, Canada; b Research Institute, Hospital for Sick Children, University of Toronto, Toronto, ON, Canada; c School of Translational Medicine, Monash University, Melbourne, VIC, Australia

**Keywords:** predicted values, pulmonary function tests, reference equations, spirometry

## Abstract

**Background:**

In 2012, the Global Lung Function Initiative (GLI) collected a large international database of spirometry measurements in healthy people from which predicted spirometric values were derived based on sex, age, and height for people from 4 different geographic ancestral groups. In 2023, a single set of predicted spirometry values was developed for the entire data set, designed to be independent of ancestry.

**Research Question:**

What impact do differences in the proportion of participants from the 4 ancestral groups at each age cohort of the GLI database have on differences between predicted values using the 2012 and 2023 equations?

**Study Design and Methods:**

Because 77.4% of participants in the GLI spirometry database are of broadly European ancestry, the GLI 2023 spirometry reference values used an inverse probability weighting to approximate an equal contribution of each of the 4 different ancestral groups to develop predicted global spirometric values. This study examined the consequences of using a constant inverse probability weighting factor for each ancestral group over the entire age range from 3.5 to 95 years.

**Results:**

The proportional number of participants from each ancestry group in each age cohort varied considerably. The contribution of the European ancestry group, which had the largest predicted lung volumes, was highest in younger children and in older adults. The net effect was that when using GLI 2023 spirometry reference values, predicted lung volumes in older adults were higher than the lung volumes predicted for any of the 4 individual ancestral groups using GLI 2012 spirometry reference values. The opposite effect was found in children and adolescents.

**Interpretation:**

It was concluded that the inverse weighting strategy used to develop the GLI 2023 spirometric reference values introduced a systematic bias that skewed the predicted values, producing progressively overestimated predicted lung volumes with increasing age in adults and progressively underestimated predicted lung volumes with increasing age in children and adolescents.

FOR EDITORIAL COMMENT, SEE PAGE 1081

**Take-Home Points**

**Study Question:** What impact do differences in the proportion of participants from the 4 ancestral groups at each age range of the Global Lung Function Initiative (GLI) database have on differences between predicted values using the 2012 and 2023 equations?

**Results:** The GLI 2023 predicted values used an inverse probability weighting to pool data from diverse groups with diverse representation by age cohort, introducing anomalous differences from GLI 2012 which have a variable effect on predicted spirometric values.

**Interpretation:** Our results show that GLI 2023 anomalously predicts a higher FVC and FEV_1_ in older adults than was observed in any of the individual ancestral groups in the GLI database and conversely underestimates the growth of FVC and FEV_1_ in children and adolescents.

Population-based spirometric reference values, primarily FEV_1_, FVC, and FEV_1_ to FVC ratio, play a crucial role in the diagnosis and management of diverse respiratory conditions. Because the FEV_1_ and FVC measured in healthy people vary with birth sex, age, and height, the use of *z* scores is recommended both for determining the presence of respiratory impairment and for assessing the severity of impairment.^1^ Although no set of spirometric reference equations represents an absolute truth for an individual person, statistically accurate reference values are essential in patient management. In this age of precision medicine, multiple factors are considered to determine the appropriate therapy for a specific patient. For the clinician to make optimal diagnostic or therapeutic decisions, the observed spirometric measurements must meet accuracy requirements and the reference values must be appropriate for the individual patient.

The interpretation of spirometry findings is very important in both the diagnosis and assessment of disease severity. For example, a value of 30% predicted for FEV_1_ has been used in the timing of lung transplantation in cystic fibrosis.^2^ Although improvement in both surgical and medical management may change the threshold to be used, the importance of an accurate predicted value is crucial. A reduction in the rate of fall of FEV_1_ has been proposed as the standard for drug efficacy.^3^ Another example is the use of the yearly decline in FVC as an important indicator of the progression of pulmonary fibrosis.^4^ The absolute yearly decline in FVC differs depending on sex, age, and height. To account for this differential impact, the yearly decline in FVC may be expressed as a percent of the person’s predicted FVC. However, both the predicted rate of change and the lower limits of normal for FVC depend on which predicted values are used. The clinician needs to be aware of how the selection of reference values affects both the predicted value and the yearly change in predicted value when assessing disease severity and progression.

In 2012, the Global Lung Function Initiative (GLI) collected data from studies designed to develop spirometric prediction equations in adults, children, or both from 72 centers in 43 countries with the goal of developing 1 set of reference values for ages 3.5 to 95 years, for male and female participants and for different geographic ancestral groups.^5^ After applying inclusion criteria, 74,187 people from the following 4 categories of ancestry, with percent of the total sample shown, were included: (1) Caucasian, 77.4%; (2) African American, 4.8%; (3) Northeast Asian, 6.7%; and (4) Southeast Asian, 11.1%. A fifth group, Other, was added to accommodate those whose ancestry was not 1 of the first 4 groups or who had either mixed or unknown ancestry. The Other category used the average of the 4 predicted values calculated for a person of the same sex, age, and height from each ancestral group. We refer to these reference values with the labels European in place of Caucasian and African in place of African American. The primary difference in the GLI 2012 spirometric prediction equations among the 4 ancestral groups is in the coefficients for the contribution of standing height to the calculation of predicted lung volume.^6^

In 2023, GLI took a different approach with the goal of developing spirometric reference values that did not require the person being tested to be assigned to any specific geographic ancestral group.^7^ Rather than using the composite Other category from GLI 2012 values, it was decided to use an inverse probability weighting in an attempt to have each ancestral group contribute equally to the predicted values. The American Thoracic Society and the European Respiratory Society recommend using the GLI 2023 values.^8^ In this study, we examined the impact of the GLI 2023 methodology for pooling data from the 4 GLI 2012 ancestral data sets on the predicted lung volumes and the predicted yearly rate of change in lung volumes.

## Study Design and Methods

Predicted FVC, FEV_1'_ and FEV_1_ to FVC ratio values using the GLI 2012^5^ and GLI 2023^7^ reference sets were generated for female and male participants of the 50th percentile height over the age range of 3.5 to 19 years,^9^ the average adult height being 163 cm for female participants and 177 cm for male participants. The predicted FVC, FEV_1_, and FEV_1_ to FVC ratio were compared between GLI 2012 and GLI 2023 values for female and male participants. In adults, the predicted annual absolute decline in FVC and the annual decline in FVC as a percent of predicted FVC also were compared between GLI 2012 and GLI 2023 values. The inverse probability weighting used to generate GLI 2023 predicted values was analyzed by age cohort separately for female and male participants. Because all data were generated from reference equations, institutional review board approval was not required.

## Results

### Adults

**Figure 1:**
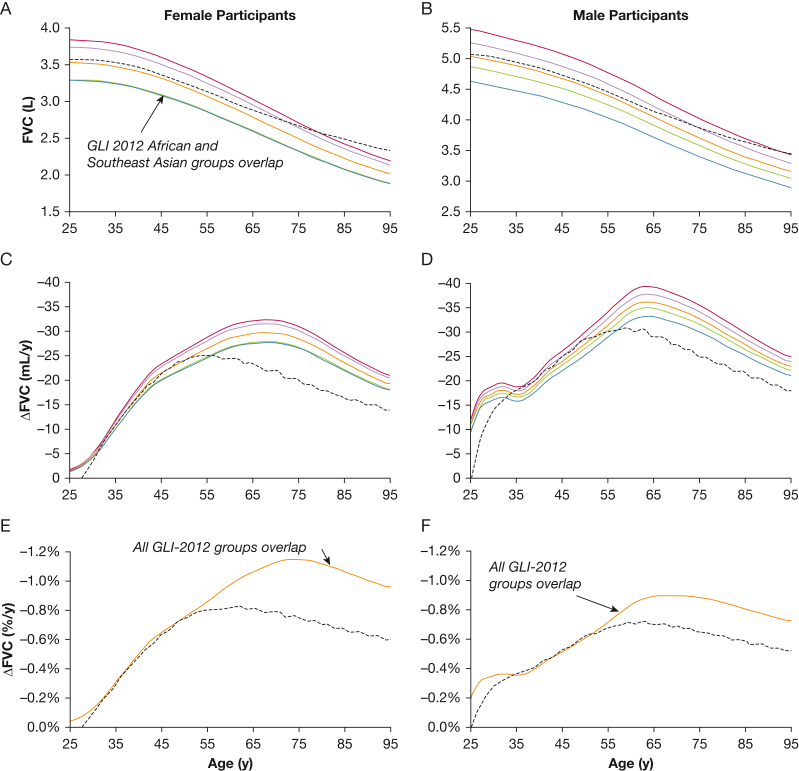
A-F, Graphs showing predicted FVC (A, B), the predicted annual decline in FVC in milliliters per year (C, D), and the predicted annual decline in FVC as a percent of predicted FVC (E, F) by female participants (A, C, E) and male participants (B, D, F) as a function of age using GLI 2023 values^7^ (dotted black line) and GLI 2012^5^ ancestral groups: European (red line), African (blue line), Northeast Asian (mauve line), Southeast Asian (green line), and Other (brown line). Overlapping lines are noted in the graphs. ΔFVC = change in FVC; GLI = Global Lung Function Initiative.

In Figure 1, the upper panels show that the predicted FVC using the GLI 2023 reference values declined more slowly with age than each of the GLI 2012 ancestral groups, which all follow a similar decline. Unexpectedly, after 78 years of age in female participants and after age 91 years of age in male participants, the predicted value for FVC using GLI 2023 values is higher than the predicted FVC for each of the 4 GLI 2012 ancestral groups. Therefore, the GLI 2023 annual decline in the predicted FVC is lower than that of all GLI 2012 ancestral groups by 55 years of age in female participants and by age 57 years of age in male participants (Figs 1C, 1D). For the annual decline in FVC as a percent of the predicted FVC (Figs 1E, 1F), all 4 GLI 2012 ancestral groups and the composite Other group overlap, with each showing the same relative rate of decline over the entire adult age range. In contrast, compared with GLI 2012 values, the GLI 2023 reference values predict a lower relative rate of annual decline in FVC starting at 52 years of age for both female and male participants. Averaged over the older range of 65 to 95 years, the relative rate of yearly decline in FVC as a percent of the predicted FVC using GLI 2023 values is 35% lower than when using GLI 2012 values in female participants and 26% lower in male participants (Figs 1E, 1F).

### Children and Adolescents

**Figure 2:**
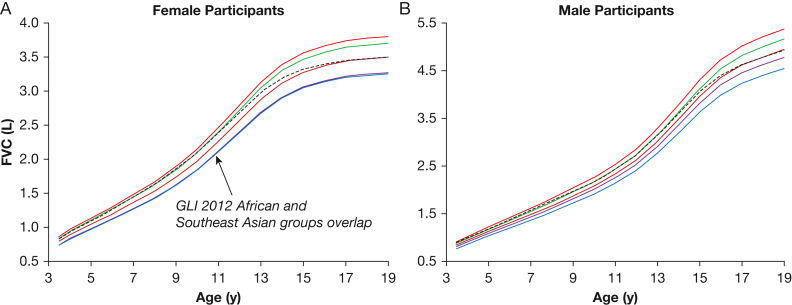
A, B, Graphs showing predicted FVC as a function of age in the pediatric range by female participants (A) and male participants (B) using GLI 2023 data^7^ (dotted black line) and the GLI 2012^5^ ancestral groups: European (red line), African (blue line), Northeast Asian (mauve line), Southeast Asian (green line), and Other (brown line). Overlapping lines are noted in the graphs. GLI = Global Lung Function Initiative.

Figure 2 shows that although the FVC predicted for each ancestral group using GLI 2012 values followed a similar rate of increase with age, the GLI 2023 values did not. The GLI 2023 predicted values were nearly equal to the GLI 2012 predicted values for the European group from 3.5 to 10 years of age in female children (Fig 2A) and from 3.5 to 8 years in male children (Fig 2B). Thereafter, the predicted growth in FVC was less using GLI 2023 values such that at 19 years of age, FVC was lower by 300 mL (8%) than the GLI 2012 predicted value for FVC in the European category in female participants and by 450 mL (8.4%) in male participants.

### Overall

To determine the separate effect of height on the difference between GLI 2012^5^ and GLI 2013^7^ predicted values, this analysis was repeated using the 25th and 75th percentiles for height. In adults, the age at which the predicted FVC using GLI 2023 values anomalously exceeded the predicted FVC for all groups using GLI 2012 values was lower for taller people (e-Table 1). The difference between GLI 2023 and GLI 2012 values regarding the decline in FVC with age as a percent of FVC predicted was unaffected by height.

The converse was found in children and adolescents. At 3.5 years of age, FVC predicted using GLI 2023 values is within 0.03 L of FVC predicted using GLI 2012 values for European ancestry. By 19 years of age, the FVC predicted using GLI 2023 values drops to less than FVC predicted using GLI 2012 values, indicating that GLI 2023 values predict a diminished rate of growth in FVC compared with GLI 2012 values. The amount of reduction in predicted FVC varies with height, with a greater reduction for those in the 25th height centile and less for the 75th height centile (e-Table 1).

Similar trends to those shown for FVC in Figures 1 and 2 were found for FEV_1_ and FEV_1_ to FVC ratio, as seen in e-Figures 1, 2, 3, and 4. The FEV_1_ was somewhat less affected than FVC. The 2 main reasons for a reduced impact are that FEV_1_ is smaller than FVC and that the GLI 2012 predicted values for FEV_1_ are distributed more tightly among the 4 ancestry groups. However, a decreased relative rate of decline of FEV_1_ with increasing age continued in adults and a decreased relative rate of growth of FEV_1_ with increasing age in children continued compared with GLI 2012 values. The FEV_1_ to FVC ratio showed less variation among the 4 ancestral groups using GLI 2012 values. Nonetheless, in adults, the predicted FEV_1_ to FVC ratio using GLI 2023 values exhibited a gradual increase with age compared with GLI 2012 values (e-Figs 1B, 1C, 3B, 3C). In children and adolescents, the FEV_1_ to FVC ratio difference was greater. The predicted value for FEV_1_ to FVC ratio using GLI 2023 values was lower than that predicted using GLI 2012 values for each of the ancestral groups from 15 to 17 years of age in male children and from 14 to 16 years of age in female children (e-Figs 2B, 2C, 4B, 4C).

## Discussion

**Figure 3:**
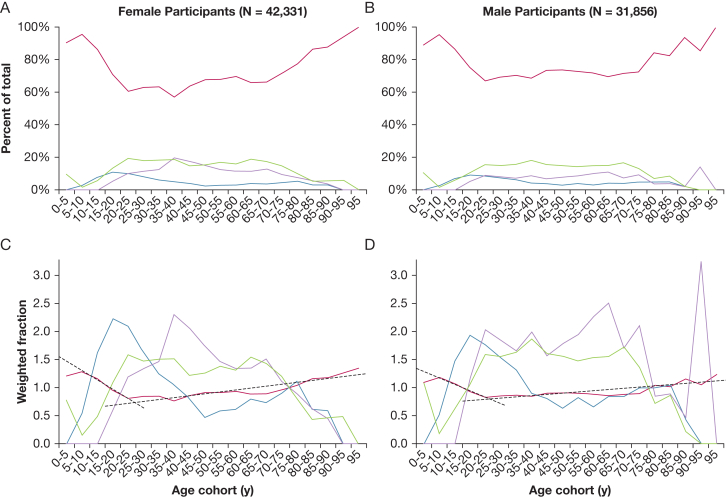
A-D, Graphs showing the distribution of the 4 ancestral groups in the Global Lung Function Initiative (GLI) data set—European (red line), African (blue line), Northeast Asian (mauve line), and Southeast Asian (green line)—by age cohort and according to female participants (A, C) and male participants (B, D). The percent of the total sample for each ancestry group (A, B) and the relative inverse probability weighted contributions used in GLI 2023^7^ (C, D) are shown. For each of the ancestral groups, the inverse probability weighted fraction, when averaged over the entire age range, is equal to 1. The dashed black lines in (C, D) are the linear regression lines for the weighted fraction of the European group vs the age cohort calculated separately for 3.5 to 19 years (decreasing) and for 20 to 95 years (increasing).

Both the GLI 2012 and GLI 2023 reference values were generated using the same database of spirometric measurements from 74,187 people 3.5 to 95 years of age. The distribution of the GLI database by ancestral group is: European, 57,394 people; African, 3,545 people; Northeast Asian, 4,991 people; and Southeast Asian, 8,255 people. As shown in the Figure 3, the proportion of the 4 ancestral groups varied with age. The European ancestral group was dominantly overrepresented, especially in the 3.5- to 20-year age range and the 65- to 95-year age range (Fig 3). The GLI 2023 reference values used the same database, but assigned an inverse probability weighting to each ancestral group by sex, based on the total number in each ancestral group as a fraction of the total number of the entire sample.^10^ Although this inverse probability weighting resulted in each ancestral group contributing equally to the predicted values when averaged over the entire range of age cohorts, considerable differences were noted in the relative contributions of the ancestral groups at different ages, as seen in Figures 3C and 3D. The Northeast Asian group includes no data from participants younger than 10 years, which results in an overweighting for this group in older age cohorts.

This indicates that the inverse probability weighting used by GLI 2023 did not neutralize the effects of different ancestry on predicted spirometric values because the relative contributions of the ancestral groups to the predicted values varied with age. In adults, the weighted contribution of the European ancestral group rose continuously from 25 to 95 years of age, as seen by the dashed black linear regression lines in Figures 3C and 3D. Conversely, in children, the weighted contribution of the European ancestral group declined from 3.5 to 19 years (Figs 3C, 3D, dashed black line). This seems to have caused the age spline calculated using the general additive models for location scale and shape technique to underestimate the growth in lung volume with age in children and to underestimate the progressive decline in lung volume with age in adults because the European ancestral group, who showed the highest lung volumes, made up more of the inverse probability weighted sample at younger ages in children and older ages in adults. The effect in adults was more pronounced in female participants than in male participants. Two potential explanations exist for this. First, almost twice as many female as male participants of European ancestry were in in the 60- to 95-year age range (24,521 vs 14,150). Second, a spike in the weighted contribution of the Northeast Asian ancestral group for male participants in the 90- to 95-year age cohort is present, which was entirely the result of 1 person. The weighting for Northeast Asian ancestry was 18.4 times higher than the weighting for European ancestry.

Clinical analysis of the change in FVC should consider the natural decline that occurs in adults in the absence of any disease process, which varies with sex, height, and age. Using the annual decline in FVC as a percent of predicted FVC will eliminate the height bias, but the sex and age bias will continue. Using the annual decline in FVC *z* score addresses all 3 potential biases. However, when using GLI 2023 *z* scores, the weighted contribution of the various ancestral groups varies with age, which results in an anomalously lower predicted annual relative decline in FVC, and consequently progressively lower FVC *z* scores, with increasing age in adults, especially those older than 55 years.

The 8- to 19-year age range shows a lower relative increase in predicted FVC using GLI 2023 values, resulting in an anomalous decrease in the lower limit of normal with increasing age. Also, differences in the rate of change in FEV_1_ to FVC ratio were noted (e-Figs 2, 4). Because the FEV_1_ to FVC ratio is a sensitive indicator of early lung disease, primarily asthma and cystic fibrosis, differences in the predicted yearly change in FEV_1_ to FVC ratio, and corresponding *z* score differences, may impact treatment and management decisions for a given individual.

Ironically, the annual decline in FVC as a percent of the predicted FVC using GLI 2012 values is truly neutral with respect to ancestry because it was observed to be the same for all 4 of the ancestral groups, whereas differences in the age-dependent inverse probability weighting used for GLI 2023 values introduced an ancestry dependence for the decline in FVC as a percent of the predicted FVC because of the progressively increased weighting of the European ancestral group with age in adults and the progressively decreased weighting of the European ancestral group in children.

This study highlights some of the limitations of the GLI data sets. A need exists for more data in the oldest age ranges, especially given the increasing population older than 65 years. Although more recent data from China^11^ and Southern Asia^12^ are available, these studies have not been added to the GLI data set. Minimal data are available from the various regions of Africa and South America. More studies are needed.

Prediction equations and lower limits of normal will provide only a statistical probability for determining functional impairment. Prediction equations that are not appropriate for the individual being tested may result in the overdiagnosis of impairment in some and the underdiagnosis in others. The result will be the expense and risk of side effects from unneeded medication in the former and the undertreatment in the latter. The usefulness of the prediction equations will depend on how well the individual is represented in the reference group used to develop the equations in terms of age and standing height. Although it may seem advantageous to lump all ancestry groups together, the diversity of the relationship between standing height and lung volume among individuals of different ancestry may negate any potential gain.^13^ Having prediction equations that are independent of ancestry may not be achievable until measures of body structure that are more closely correlated to lung volume are validated.^6^^,^^13^

## Interpretation

The GLI 2012 spirometry reference set provides predicted values that were derived from an analysis of 4 different ancestral groups (European, African, Northeast Asian, and Southeast Asian) plus an additional Other set of reference values to be used when ancestry is either not 1 of these 4 groups, is mixed, or is unknown. The Other predicted value for a given variable is simply the average of the 4 predicted values for a person of the same sex, age, and height using the equations for each ancestral group. Although the goal of the GLI 2023 reference values was to develop spirometric predicted values that are neutral with respect to ancestry, the inverse probability weighting introduced an ancestry bias that varies with age and anomalously predicts a higher FVC and FEV_1_ in older adults than was predicted in any of the ancestral groups in the GLI data base. Conversely, the same mechanism causes an anomalous underestimation of the growth of FVC and FEV_1_ in children and adolescents. The GLI 2012 Other category provides composite results from the GLI spirometry data set that follow a similar track across age and height as observed in the 4 ancestral groups.

In conclusion, although the intent of GLI 2023 values was to develop spirometric reference equations that are independent of ancestry, the design process using statistical manipulation to pool data from diverse groups with diverse representation by age cohort introduced anomalies that have a variable effect on the interpretation of spirometry results. Using the GLI 2012 Other category to eliminate the need to assign an individual to a particular ancestral group avoids the problems incurred by using the GLI 2023 reference equations.

## Funding/Support

V. M. is supported by the University of Saskatchewan and the Royal University Hospital Foundation. Y. H. K. is supported by an investigator grant from NHMRC Australia, MRFF, the Austin Medical Research Foundation, and the Lung Foundation Australia.

## Financial/Nonfinancial Disclosures

The authors have reported to *CHEST* the following: B. L. G. reports consulting fees from AstraZeneca Global, ArtiQ (Clario), Merz Pharmaceuticals, UCLA Medical School, and the Saskatchewan Health Authority; speaker honoraria from LungSask, Lung Association of Nova Scotia and Prince Edward Island, and Canadian Association of Cardio-Pulmonary Technologists; travel and accommodation expenses for conference attendance from Novus Medical, Inc.; and a patent agreement with Hans Rudolph, Inc. V. M. reports personal fees from Boehringer Ingelheim, Hoffman-La Roche, Ltd., and Astra Zeneca; and grants from the University of Saskatchewan, Royal University Hospital Foundation, Boehringer Ingelheim, Astra Zeneca, and Hoffman-La Roche. Y. H. K. reports fellowship support from an investigator grant from NHMRC, Australia and grant funding from MRFF, Austin Medical Research Foundation, and Lung Foundation Australia. A. L. C. reports an honorarium as a visiting professor from Manidol University, Bangkok, Thailand.

## Supplementary Data

e-Online Data
