# Supplementary material for: Anomalous Differences Between the Global Lung Function Initiative 2023 and 2012 Spirometry Reference Values
Source: Chest. 2025 Jun 17;168(5):1178–85. doi: 10.1016/j.chest.2025.06.005 (PMC12831081; doi:10.1016/j.chest.2025.06.005)
Supplement: e-Online Data [file mmc1.pdf]

Supplemental materials for: Anomalous differences between the Global Lung Function Initiative 2023 and 2012 spirometry reference values.
Graham BL, Marcoux V, Khor YH, Coates AL.

Pages 2 to 5: predicted values for FEV_1, FVC and FEV_1/FVC using GLI-2023 and GLI-2012 over the age range of 3.5 to 95 years.
Graphs are provided separately for:

- e-Figure 1. Females age 20 to 95 years, height 163 cm
- e-Figure 2. Females age 3.5 to 20 years, 50th percentile height with adult height of 163 cm
- e-Figure 3. Males age 20 to 95 years, height 177 cm
- e-Figure 4. Males age 3.5 to 20 years, 50th percentile height with adult height of 177 cm

Page 6:
e-Table 1. The effect of height on differences between FVC predicted using GLI-2023 compared to GLI-2012.

Predicted values using GLI-2023, also called GLI Global, are derived from:
Bowerman C, Bhakta NR, Brazzale D et al. A Race-neutral Approach to the Interpretation of Lung Function Measurements, *Am J Respir Crit Care Med*. 2023;207(6):768-774. doi: 10.1164/rccm.202205-0963OC.

Predicted values using GLI-2012 are derived from:
Quanjer P, Stanojevic S, Cole T, et al. Multi-ethnic reference values for spirometry for the 3-95-yr age range: the global lung function 2012 equations. *Eur Respir J.* 2012;40(6):1324-1243. doi: 10.1183/09031936.00080312.

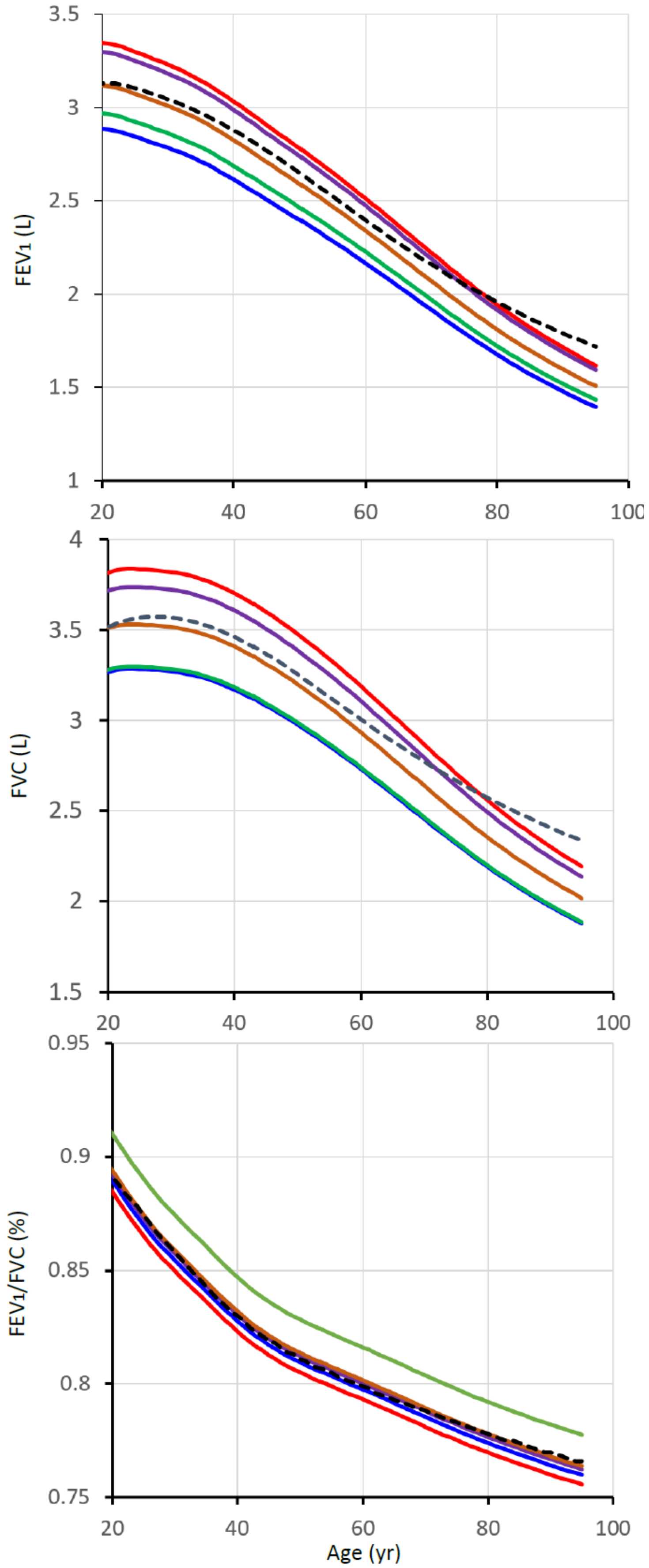

e-Figure 1

Predicted spirometric variables in adult females from age 20 to 95 years of 163 cm in height.

Legend:
GLI-2023 (dashed black lines)

GLI-2012 ancestral groups:
- European (red lines)
- African (blue lines)
- NE Asian (mauve lines)
- SE Asian (green lines)
- Other (brown lines)

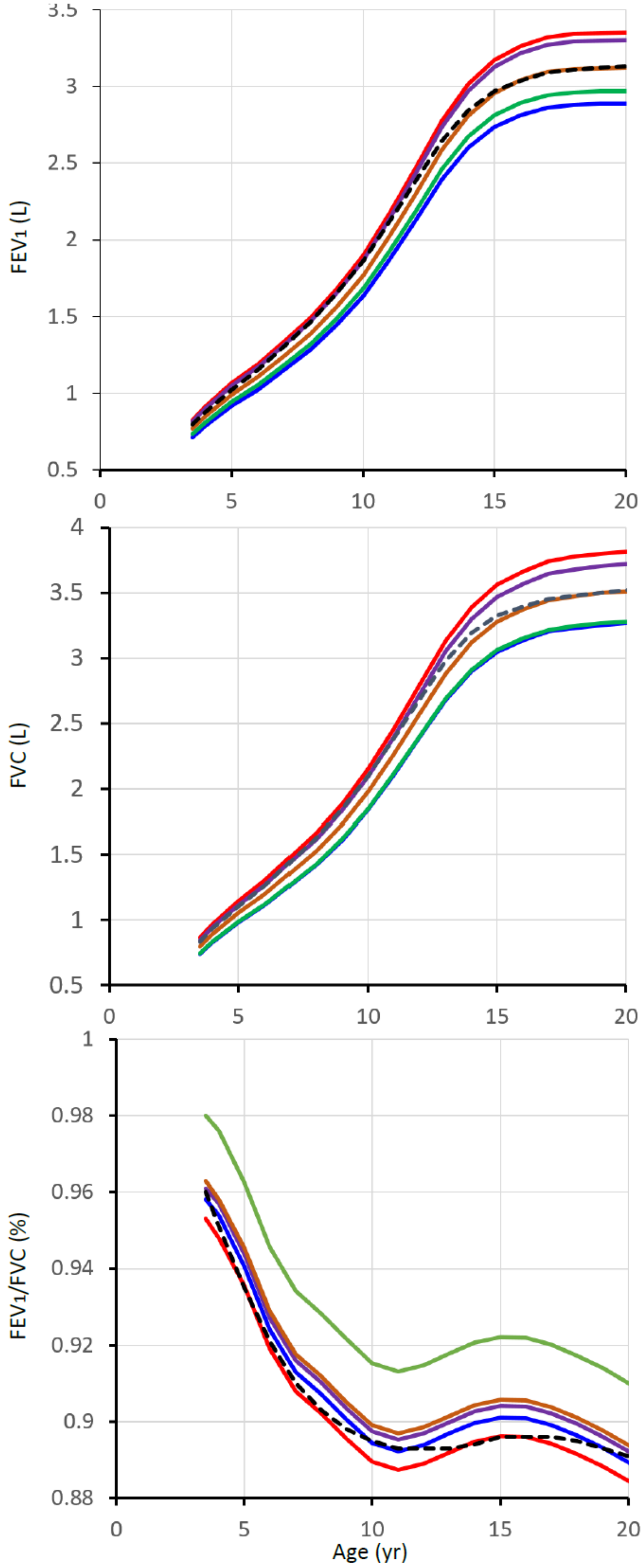

e-Figure 2

Predicted spirometric variables in childhood and adolescent females from age 3.5 to 20 years of median height.

Legend:
GLI-2023 (dashed black lines)

GLI-2012 ancestral groups:
- European (red lines)
- African (blue lines)
- NE Asian (mauve lines)
- SE Asian (green lines)
- Other (brown lines)

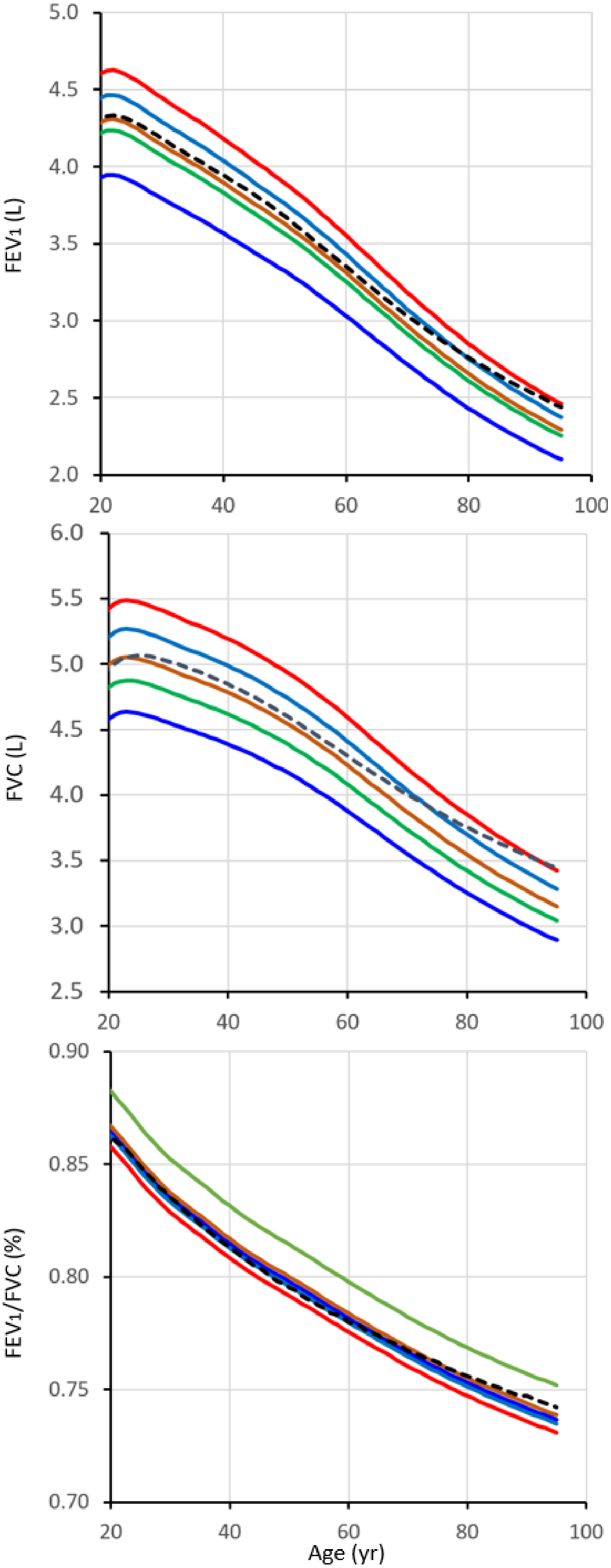

e-Figure 3

Predicted spirometric variables in adult males from age 20 to 95 years of 177 cm in height.

Legend:
GLI-2023 (dashed black lines)

GLI-2012 ancestral groups:
- European (red lines)
- African (blue lines)
- NE Asian (mauve lines)
- SE Asian (green lines)
- Other (brown lines)

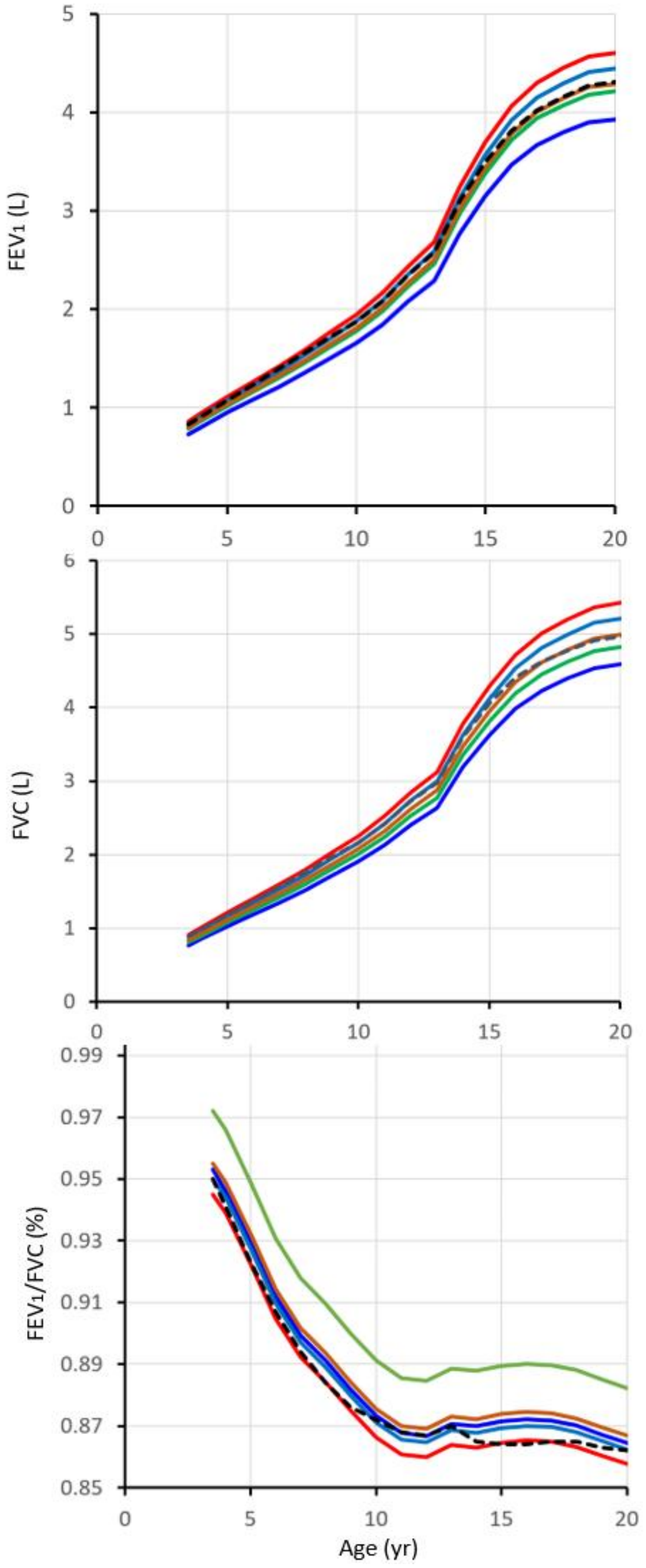

e-Figure 4

Predicted spirometric variables in childhood and adolescent males from age 3.5 to 20 years of median height.

Legend:
GLI-2023 (dashed black lines)

GLI-2012 ancestral groups:
- European (red lines)
- African (blue lines)
- NE Asian (mauve lines)
- SE Asian (green lines)
- Other (brown lines)

Tables

e-Table 1. The effect of height on differences between FVC predicted using GLI-2023 compared to GLI-2012.

**Sex**	**Females**			**Males**		
**Height percentile**	25th	50th	75th	25th	50th	75th
**Age of FVC divergence[1]**	81 yr	79 yr	77 yr	>95 yr	92 yr	86 yr
**Difference in FVCpred at age 19 yr[2]**	0.315 L 8.8%	0.302 L 7.9%	0.284 L 7.0%	0.464 L 9.7%	0.448 L 8.4%	0.424 L 7.2%

[1]The age at which FVC predicted using GLI-2023[7] exceeds the FVC predicted using GLI-2012[5] for all of the ancestral groups in the GLI dataset.
[2]FVC predicted using GLI-2012 - FVC predicted using GLI-2023. At age 3.5 the differences are all within 0.03 L, but later in childhood, the GLI-2023 FVC is progressively lower than GLI-2012, becoming 7.0% to 9.7% lower by age 19.
